# Two-Photon Microperimetry: A Media Opacity-Independent Retinal Function Assay

**DOI:** 10.1167/tvst.10.2.11

**Published:** 2021-02-10

**Authors:** Ang Wei, Urmi V. Mehta, Grazyna Palczewska, Anton M. Palma, Vincent M. Hussey, Luke E. Hoffmann, Anna Diep, Kevin Nguyen, Bryan Le, Steven Yone-Shun Chang, Andrew W. Browne

**Affiliations:** 1 UCI Health Gavin Herbert Eye Institute, Department of Ophthalmology, University of California-Irvine, Irvine, CA, USA; 2 College of Osteopathic Medicine of the Pacific, Western University of Health Sciences, Pomona, CA, USA; 3 Department of Medical Devices, Polgenix, Inc., Cleveland, OH, USA; 4 Institute for Clinical and Translational Sciences, University of California-Irvine, Irvine, CA, USA; 5 UCI School of Medicine, University of California-Irvine, Irvine, CA, USA; 6 Creighton University School of Medicine, Omaha, NE, USA; 7 Drexel University College of Medicine, Philadelphia, PA, USA; 8 Department of Biomedical Engineering, University of California-Irvine, Irvine, CA, USA

**Keywords:** two-photon microperimetry, infrared light, retinal sensitivity, microperimetry, simulated media opacities

## Abstract

**Purpose:**

Compare results obtained using infrared two-photon microperimetry (2PM-IR) with conventional visual function tests in healthy subjects of varying ages with and without simulated media opacities.

**Methods:**

Subjects from two separate cohort studies completed cone contrast threshold (CCT) testing, conventional microperimetr*y*, visible light microperimetry from a novel device (2PM-Vis), and infrared two-photon microperimetry. The first cohort study, which consisted of six healthy volunteers (23 to 29 years of age), evaluated the effects of simulated media opacities on visual performance testing. Subjects underwent testing on four visual function devices nine separate times under the following conditions: no filter, red filter, green filter, blue filter, light brown filter, dark brown filter, polarized black filter (0° rotation), and polarized black filter (90° rotation). Subjects subsequently performed 2PM-IR and 2PM-Vis testing without a filter in the mydriatic state. The second cohort study evaluated the effect of age on visual test performance in 42 healthy subjects split between two groups (ages 20–40 years and 60–80 years).

**Results:**

Retinal sensitivity measured by 2PM-IR demonstrated lower variability than all other devices relying on visible spectrum stimuli. Retinal sensitivity decreased proportionally with the transmittance of light through each filter. CCT scores and retinal sensitivity decreased with age in all testing modalities. Visible spectrum testing modalities demonstrated larger test result differences between young and old patient cohorts; this difference was inversely proportional to the wavelength of the visual function test.

**Conclusions:**

2PM-IR mitigates media opacities that may mask small differences in retinal sensitivity when tested with conventional visual function testing devices.

**Translational Relevance:**

Conventional visual function tests that emit visible light may not detect differences in retinal function during the early stages of age-related diseases due to the confounding effects of cataracts. Infrared light, which has greater transmittance through ocular tissue, may reliably quantify retinal sensitivity and thereby detect degenerative changes early on.

## Introduction

Visual function is fundamental to eye care. Accurate measurements can identify visual disorders and prompt medical intervention to preserve and restore vision. The highest acuity and color vision are subserved by the fovea located centrally in the light sensitive retina. The fovea is densely populated with three types of cones that are responsible for central visual function. Cone function is evaluated subjectively with visual acuity (VA) testing,^1^^,^^2^ color and contrast testing,^3^^,^^4^ and microperimetry.^5^ All conventional tests of visual function use visible light, and their results are altered by ocular media opacities such as cataracts, corneal scars, and dense vitreous debris. A visual function test that is less susceptible to media opacity would produce more reliable information about retinal function in common blinding diseases. This would be most useful in diseases with concomitant cataract such as age-related macular degeneration, myopic degeneration, diabetic retinopathy, and glaucoma.

VA testing is the most common method for evaluating central visual function and is the gold standard for assessing functional primary outcomes in clinical trials.^1^ VA testing is often performed using the Early Treatment Diabetic Retinopathy Study (ETDRS) or Snellen charts. However, VA is a poor predictor of total visual function,^6^ especially in patients with macular disease, as it provides no information on contrast sensitivity, color vision, or metamorphopsia. Additional information for central visual function is available with cone contrast threshold (CCT) testing and microperimetry. CCT is an adaptive computer-based program that quantifies the severity and type of color vision deficiency (CVD)^3^^,^^4^ by selectively stimulating L-cones, M-cones, and S-cones. Microperimetry is a retinal sensitivity assay that provides quantitative retinal sensitivity data for both central and peripheral microscopic foci in the retina.^5^ Such data are superimposable on real-time fundus images to correlate abnormalities in retinal structure with function.

These conventional visual function tests rely on visible spectrum stimuli, typically described as ranging from 400 to 700 nm, and, like all subjective visual function tests, they suffer from diminished reliability in patients with media opacities. VA testing using ETDRS charts has proven to be a poor predictor of macular function in patients with macular edema secondary to retinal vascular disease, in part due to anterior or posterior segment opacities.^7^ Cataractous lenses decrease the sensitivity threshold of all three cone classes due to increased scatter and decreased transmittance of visible light,^8^^–^^11^ with the greatest impact on short-wavelength cones, especially after the age of 60.^11^^,^^12^ Because of this effect, Fujikawa et al.^13^ established that CCT can be used reliably up to the seventh decade of life and after cataract surgery in elderly patients. Cataractous lenses and posterior capsular opacification^14^ also diminish retinal sensitivity measurements obtained using microperimetry. This decrease is dependent on the density and type of cataract formed.^6^

Boettner and Wolter^15^ showed that scattering decreases and transmittance increases as the wavelength elongates from visible to infrared (IR) ranges. Therefore, if humans perceived IR light, visual function testing would be less affected by cataracts and other media opacities. Previously, a two-photon (2P) microperimeter demonstrated that IR laser light can initiate phototransduction when visual pigments absorb two-photon light, inducing photoisomerization of 11-*cis*-retinal (opsins in rod and cone photoreceptors).^16^^,^^17^ Therefore, 2P IR stimulation of the retina provides an opportunity to evaluate retinal function and quantify visual function in a manner less susceptible to media opacity.

To explore this topic, a two-photon microperimeter using pulsed IR light (2PM-IR) at 1045 nm was compared with US Food and Drug Administration (FDA)-approved clinical visual function devices: CCT and conventional microperimetry (cMP). The 2PM device can also produce a visible 522.5-nm stimulus (2PM-Vis), which was also compared with 2PM-IR. Although conventional microperimetry devices rely on automated retinal tracking, the prototype 2PM device relies on the operator positioning the visual stimulus on a specific point of a scanning laser ophthalmoscopy image acquired in real time. Therefore, manual tracking was used to achieve microperimetric testing. Young healthy volunteers performed testing on all four devices using different optical filters that were placed directly in front of their eyes. A second analysis was performed in two separate cohorts comparing the effect of age on test performance using conventional and novel visual function tests. 2PM-IR visual stimulation was hypothesized to produce visual sensitivity results that are more consistent when tested in healthy subjects of all ages with and without filters than the three visible light visual function assays: CCT, cMP, and 2PM-Vis.

## Methods

This study received institutional review board approval from the University of California-Irvine and was conducted in accordance with the tenets of the Declaration of Helsinki. All participants provided written informed consent before testing began. Two prospective studies evaluated the effects of various testing conditions and age on test performance.

### Subjects and Protocol

#### Effects of Filters and Mydriasis on Visual Function Testing

**Figure 1.**
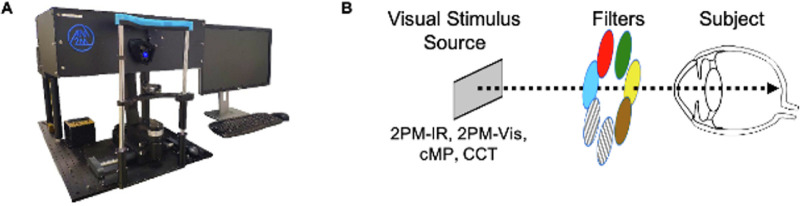
(**A**) 2PM is capable of testing retinal sensitivity using single-photon visible (2PM-Vis) and two-photon infrared (2PM-IR) stimuli. (**B**) Configuration for measuring cone contrast/retinal sensitivity under the nine different testing conditions.

The effects of various testing conditions on test performance were evaluated in this prospective study. Six phakic eyes of six healthy subjects (four males, two females; ages 23–29 years) underwent visual function testing using four different visual function devices: CCT (ColorDx CCT HD; Konan Medical USA, Irvine CA), cMP (MP-3; Nidek, Inc., San Jose, CA), 2PM-Vis, and 2PM-IR. Figure 1a displays the novel 2PM apparatus for measuring retinal sensitivity. Nine separate tests were performed on each device under the following conditions: no filter, red filter, green filter, blue filter, light brown filter, dark brown filter, polarized black filter (0° rotation), and polarized black filter (90° rotation) (Fig. 1b). Subjects subsequently performed 2PM-IR and 2PM-Vis testing without a filter in the mydriatic state following instillation of 0.75% tropicamide and 2.5% phenylephrine.

#### Effects of Age on Test Performance

The effects of age on test visual function were evaluated in 42 healthy subjects. Nineteen subjects (33 eyes) between the ages of 20 and 40 years and another 23 subjects (32 eyes) between the ages of 60 and 80 years were analyzed using CCT, 2PM-Vis, and 2PM-IR. Inclusion criteria for both analyses were (1) age > 18 years, (2) no history of ocular disease, (3) best-corrected visual acuity (BCVA) of 20/25 or better, and (4) phakic lens status. Normal ophthalmic health was determined by clinical examination by a retina specialist and review of color fundus photography (Clarus; Carl Zeiss Meditec, Jena, Germany) and spectral-domain optical coherence tomography (Cirrus 5000, Carl Zeiss Meditec) demonstrating normal anatomy. Visual acuity was measured following ETDRS testing protocol.

#### Spectral Emission and Transmission

The spectral transmission of each filter was measured using a compact spectrometer (CCS200/M, Thorlabs, Newton, NJ) in indirect sunlight and quantified from 350 to 900 nm. Filter transmission at 1045 nm was quantified using a 1045-nm laser source. The spectral emission of RGB light from the ColorDx liquid-crystal display (LCD) was measured using the same compact spectrometer. The spectral emission from cMP was not obtained due to difficulty with the probing light in the device oculars but is assumed to be similar to the ColorDx LCD.

### Functional Testing

#### Two-Photon Microperimetry

2PM (Polgenix, Inc., Cleveland, OH) (Fig. 1a) is a custom apparatus that is the product of the National Eye Institute's Audacious Goal Initiative to develop new technology for the non-invasive assessment of retinal structure and function in vivo. 2PM is a retinal sensitivity assay capable of stimulating the retina using pulsed two-photon IR light at 1045 nm (2PM-IR) or standard single-photon visible green light at 522.5 nm (2PM-Vis) to evaluate foveal sensitivity. Testing was performed in a dimly lit room using the subject's BCVA after 30 minutes of dark adaptation. A stimulus pattern formed either by two-photon infrared or visible light was projected onto the retina as previously described.^17^ Briefly, a pulsed two-photon IR signal was generated as a 63-MHz pulse train of 250 fs-long pulses with a 1045-nm central wavelength. Visible (green) light at 522.5 nm was produced by diverting the IR light source to a nonlinear crystal, producing a half-wavelength change of the 1045-nm laser source to a 522.5-nm visible stimulus.^17^ Test subjects were asked to adjust the intensity of the stimulus by scrolling either up or down on a computer mouse, which would either increase or decrease the intensity of the stimulus entering the eye. When the visibility threshold was reached and the subject could no longer see the stimulus, the subject was asked to left click on the mouse, thereby recording the threshold power that could be detected. The same procedure was performed four more times, and an average threshold sensitivity for all five replicate tests was calculated. Small refractive errors were corrected using correction lenses internal to the 2PM.

2PM-IR and 2PM-Vis stimulus intensity are measured in microwatts and femtowatts, respectively. Retinal sensitivity (S) was calculated using the inverse of the average threshold power (S = 1/threshold power). This value (*P*_1_) was then converted to decibels using 100 pW (*P*_0_) of threshold power as the reference value using the following equation:^17^

$$\begin{array}{c} \mathrm{dB}=10\;\log\left(P_{1}/P_{0}\right) \end{array}$$

#### Conventional Visible Microperimetry

Conventional microperimetry was performed using the MP-3, a clinical microperimetry assay used to quantify macular sensitivity. It was performed only for the foveal point under photopic conditions without correction. The contralateral eye was occluded. Goldmann III stimuli were applied over an intensity range from 0 to 34 dB. An IR sensor was used to track eye movement, and a white light-emitting diode light source was used to project a stimulus pattern onto the retina.

#### Cone Contrast Threshold Testing

The ColorDx CCT HD is an adaptive visual function testing device that selectively stimulates retinal L-, M-, and S-cones. Landolt C optotypes are projected in either decreasing or increasing steps of cone contrast against an isoluminant gray background.^19^ Subjects indicate the orientation of the gap in the Landolt C optotype using arrows on a trackpad. The final score was calculated based on the number of correct answers. CCT was performed monocularly using the subject's BCVA under photopic conditions at a distance of 2 feet. Score reports are as follows: >90 is normal, 75 to 90 indicates possible CVD, and <75 indicates CVD.

### Statistical Analysis

Mean sensitivities and standard deviation were calculated for each test. Paired-sample *t*-tests were performed to assess whether mydriasis affected retinal sensitivity. Linear regression models were fit to compare the effect of age between young and old subjects on visual function test performance assessed using CCT, cMP, 2PM-Vis, and 2PM-IR. Standardized coefficients and 95% confidence intervals (CIs) were used to estimate the effect size between young and old age groups for each testing modality, as unit scales varied among the devices. Standard coefficients can be interpreted as the differences in standard deviations between young and old groups. Test score distributions by age group are illustrated using violin plots. All statistical analyses were performed using SPSS Statistics 25 (IBM, Armonk, NY) and R 3.6.1 (R Foundation for Statistical Computing, Vienna, Austria).

## Results

### Filter Spectral Transmission and CCT Screen Emission

**Figure 2.**
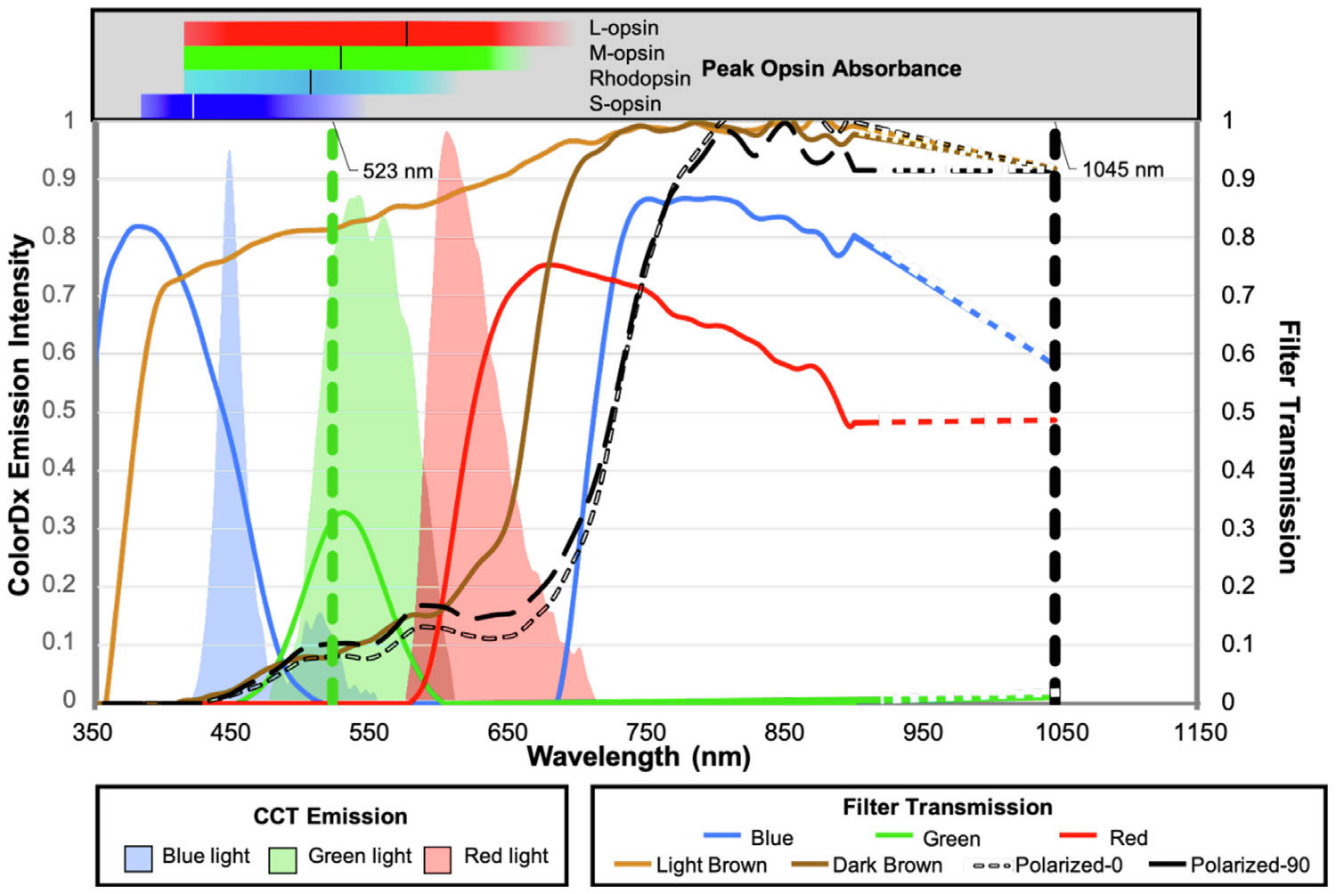
Spectral transmission of filters (lines) superimposed on the spectral emission of RGB light from the CCT monitor (shaded regions). Horizontal bars above the plot represent opsin spectral absorbances, with *vertical lines* highlighting peak spectral absorption.^18^ Vertical dashed lines indicate wavelengths of visible green 2PM-Vis (523 nm) and pulsed infrared 2PM-IR (1045 nm) stimulation.

Figure 2 displays the spectral emission of the CCT monitor as color-coded shaded histograms, transmission spectrum for media opacity-simulating filters as line graphs, and peak opsin spectral absorbances as horizontal bar plots. The spectral emission ranges for blue, green, and red light are 418 to 556 nm (peak, 445 nm), 475 to 615 nm (peak, 541 nm), and 577 to 724 nm (peak, 608 nm), respectively. The peak spectral absorbances for rods, S-cones, M-cones, and L-cones are: 498 nm, 420 nm, 534 nm, and 564 nm respectively.^18^

The green filter transmits the least light across the full spectrum with low transmission, even in the green spectrum centered around 531 nm and, notably, allows no transmission of IR light. The blue filter has maximal transmission in the blue spectrum centered around 379 nm and permits the transmission of some IR light. The red filter has maximal transmission in the red spectrum centered around 681 nm and permits the transmission of IR light. The light brown filter minimally attenuates shorter wavelengths in the ultraviolet–blue spectrum, whereas the dark brown and black polarized filters significantly attenuate the entire visible spectrum. The light brown, dark brown, and dark black polarized filters all permit IR transmission at 1045 nm.

### Microperimetry

**Figure 3.**
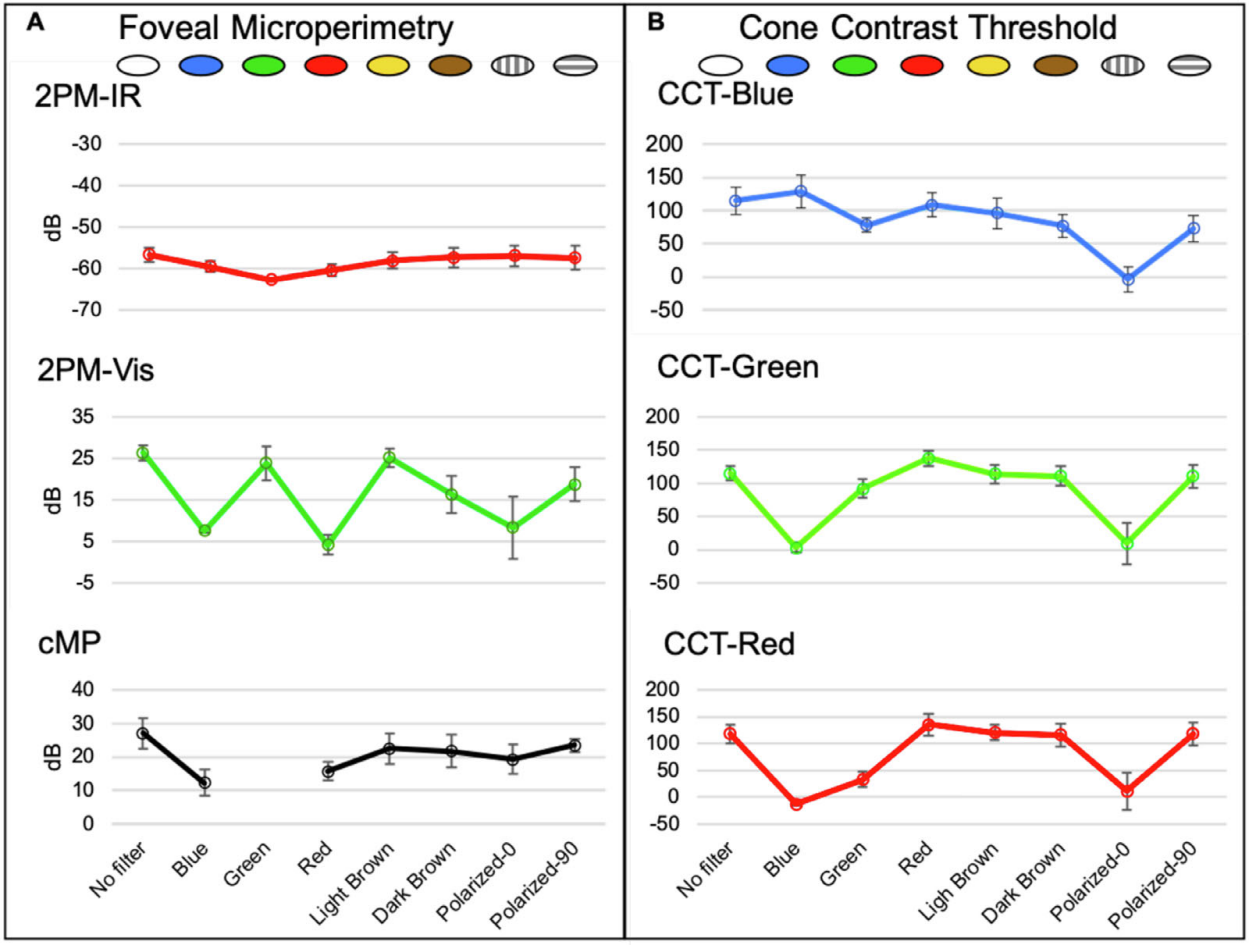
Effect of filters on visual function testing. (**A**) Foveal microperimetry with 2PM-IR, 2PM-Vis, and cMP testing. (**B**) Cone contrast threshold testing for each opsin.

**Figure 4.**
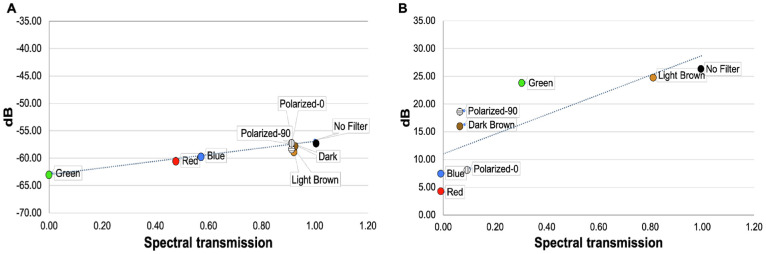
Linear regression of averaged retinal sensitivities versus filter spectral transmission for (**A**) 2PM-IR (*R*^2^ = 0.965, β = –62.967, *P* < 0.001) and (**B**) 2PM-Vis (*R*^2^ = 0.637, β = 11.002, *P* = 0.005).

Retinal sensitivity was greatest when no filter was used for all three perimetry tests (Fig. 3a). Each filter condition decreased retinal sensitivity to a degree dependent on its transmission with *P* values less than 0.001 for 2PM-IR and 0.018 for 2PM-Vis (Fig. 4). 2PM-IR sensitivity was most attenuated by the green filter and was marginally reduced by the blue and red filters. Light brown, dark brown, and black polarized filters marginally reduced retinal sensitivity. 2PM-Vis sensitivity was greatest with the green and light brown filters and lowest with the red filter. Blue and red filters severely diminished retinal sensitivity results on cMP. The green filter blocks IR light, preventing cMP from registering eye movement; therefore, no data were recorded for this filter.

### Cone Contrast Threshold

All three cones were most sensitive when their respective color filter (RGB) was applied (red filter to stimulate L-opsin, green filter to stimulate M-opsin, and blue filter to stimulate S-opsin). The shaded filters (light brown, dark brown, and dark black polarized filters) maximally reduced blue cone sensitivity, with only a nominal reduction in red and green cone sensitivities. The black polarized filter had the greatest reduction in all cone classes when oriented 0° parallel to the polarization of the CCT liquid crystal display.

### Variability of Perimetry and CCT Measures

Although CCT is not a perimetry assay and reports sensitivity as a unitless Rabin performance score,^3^ the variability in results was similar to that seen for visible light perimetry (2PM-Vis and cMP). The sensitivity ranges for cMP, 2PM-Vis, and 2PM-IR were 11.33 dB (SD, 4.96), 22.17 dB (SD, 2.14), and 5.99 dB (SD, 0.87), respectively.

### Mydriatic Versus Non-Mydriatic

The Table reports the effect of mydriasis on retinal sensitivity. Test subjects performed 2PM-Vis and 2PM-IR testing in the non-mydriatic and mydriatic states. Mydriasis had a nominally positive but statistically insignificant effect on retinal sensitivity.

**Table.** Effect of Mydriasis Using 2PM

	Mean ± SD			
	No Mydriasis (dB)	Mydriasis (dB)	Mean Difference (95% CI)	*P*
IR	–57.89 (2.31)	–56.50 (3.33)	1.39 (–0.09 to 2.87)	0.06
Vis	24.31 (5.67)	26.45 (2.03)	2.14 (–2.56 to 6.83)	0.30

Paired *t*-tests were performed to assess the effect of mydriasis on retinal sensitivity.

### Effects of Age on Test Performance

**Figure 5.**
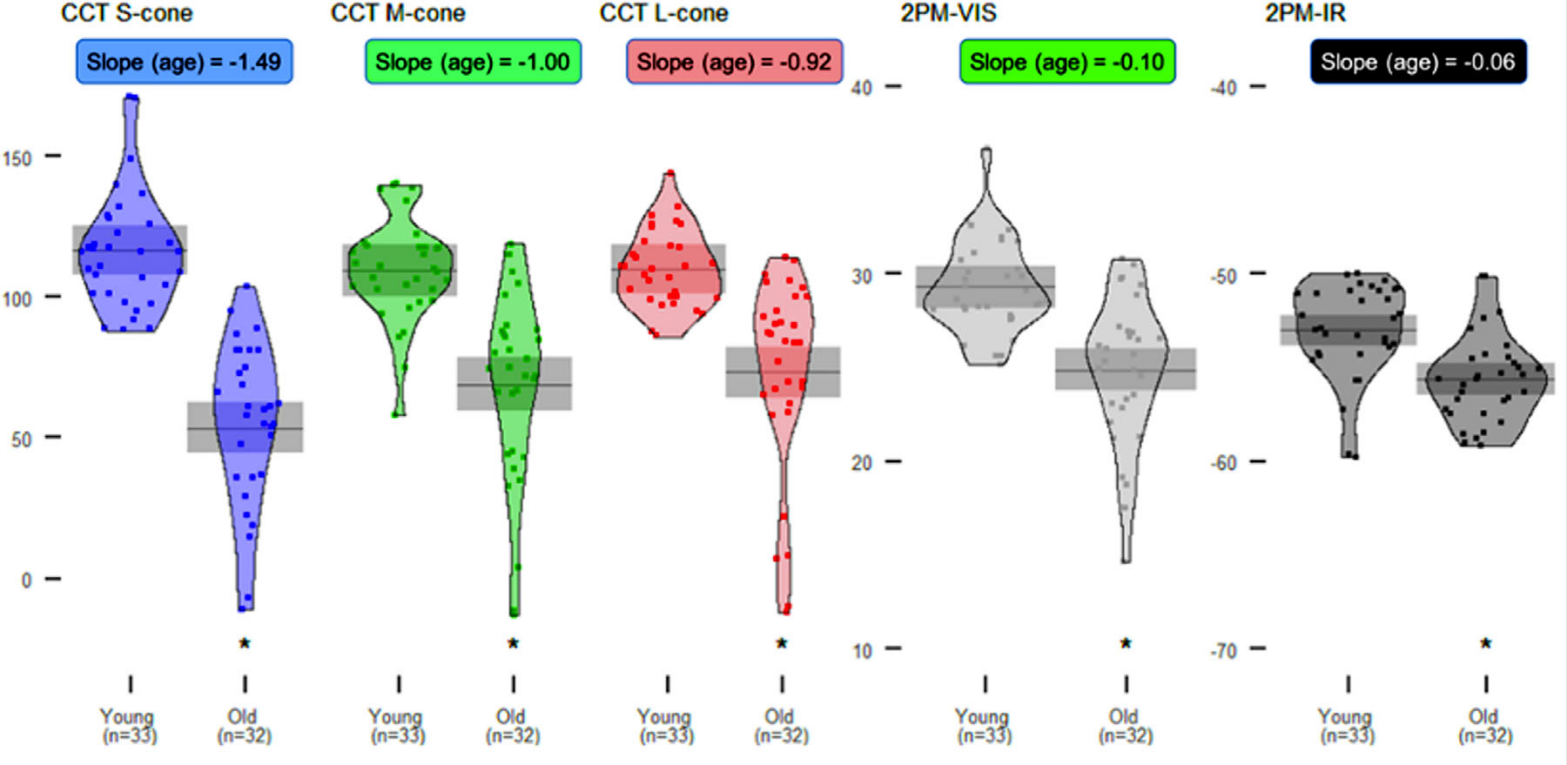
Color vision score distributions and means (95% CIs) by age. CCT scores and retinal sensitivity were compared between young (20–40 years of age) and old (60–80 years of age) healthy subjects using conventional and novel visual function testing devices. Asterisks indicate statistically significant differences (*P* < 0.05) between the young and old groups. Slope indicates decrease in score per year increase in age for each test, modeled using linear regression.

Results from visual function testing in young and old subjects are reported in Figure 5. Nineteen subjects (33 eyes) between the ages of 20 and 40 years were included in the “young” group and 23 subjects (32 eyes) between the ages of 60 and 80 years were included in the “old” group. The distributions of visual function test scores by age group are displayed using violin plots. The effect of age is represented by the means and 95% CIs for each group (gray lines and bars, respectively). CCT scores and retinal sensitivity were lower in older subjects across all testing devices. The rate of decline between young and old groups was greatest for S-cones, with a slope for each year increase in age of –1.49 (95% CI, –1.9 to –1.2) on CCT, and was least for 2PM-IR, with a slope of –0.6 (95% CI, –1.4 to –0.5). M- and S-cone function differences between the two age groups were sequentially smaller than the S-cone class and were inversely proportional to wavelength. Differences between the young and old groups were statistically significant across all testing modalities. The difference was greatest for the S-cone CCT and least for 2PM-IR. The violin plot heights were shorter and more compact for both age groups when assessed by 2PM-IR than 2PM-Vis and CCT.

## Discussion

Test performance comparing two FDA-approved visual function tests (CCT and cMP) and two novel microperimetry assays (2PM-IR and 2PM-Vis) were evaluated in two separate studies. Young healthy subjects performed testing under nine unique optical conditions. Two optical conditions were brown filter lenses. Spectral transmission of brown filter lenses with low and high brunescence were found to be comparable to published reports for aging human crystalline lens.^11^ CCT scores and retinal sensitivity were negatively affected by all seven filters; however, results obtained using 2PM-IR produced results with the least variability (Figs. 3, 4). The 2PM-IR regression plots were nearly flat and independent of visible spectrum optical transmission (Fig. 4), indicating a near independence from opacities, such as cataracts, which attenuate light in the visible spectrum. Results obtained from CCT were highly variable and dependent on media filters, highlighting that CCT may not be a reliable assessment of macular health in patients with media opacity as was previously noted.^13^

Retinal sensitivity assessed via 2PM-IR and cMP was proportional to the transmission of IR light through each filter (Fig. 3a). The green filter completely blocked transmission of IR light, thus preventing cMP from registering retinal landmarks and diminishing 2PM-IR retinal sensitivity (Figs. 2, 3a, 4a). Retinal sensitivity using 2PM-Vis was proportional to the transmission of green light through each filter (Figs. 2, 3a, 4b). The red filter permitted negligible transmission of 522.5-nm light, and subjects were unable to detect the green stimulus pattern. As anticipated, the light brown filter had the highest transmission rate at 522.5 nm and produced results with the highest retinal sensitivity.

CCT values were proportional to the amount of overlap between the spectral emission and transmission of each filter of the LCD (Fig. 2). The red filter overlaps the spectral emission of both the red and green LCD pixels, and color sensitivity was concordant for the red and green cones. The green filter produced the greatest color sensitivity for the green and blue cones, as both of these cones have overlapping absorbance curves that overlap with the green filter spectral transmission.

CCT scores and retinal sensitivity declined with age across all visual function testing devices in phakic subjects. However, the difference in score reports between young and old subjects decreased as the wavelength of the stimulus projected onto the retina increased, indicating that light in the near-IR spectrum has greater transmittance and penetrance through the aged eye than shorter wavelength light. The CCT results demonstrate the expected trend of longer wavelengths entering the eye with less attenuation, as S-cones showed the greatest difference between young and old subjects, and L-cones showed the smallest difference. Similarly, retinal sensitivity obtained using 2PM-Vis showed larger differences between the two groups compared to 2PM-IR, indicating that visual function testing using IR light is less affected by lenticular senescence and cataract formation. Furthermore, the violin plots for 2PM-IR are shorter for both age groups compared to 2PM-Vis, highlighting that IR light is less absorbed and less scattered than visible light, diminishing test variability.^17^ In this work, both 2PM-IR stimulation at 1045 nm and single-photon 2PM-Vis stimulation at 522.5 nm were perceived as a green stimulus. Because two-photon infrared stimulation results in a perceived stimulus that is half the wavelength of the IR stimulus,^16^ 2PM-IR can be implemented to test the sensitivity of each opsin. Although we performed this test under scotopic conditions, the visual sensitivity was performed exclusively in the fovea, and the perceived color to IR stimulation and visible stimulation was identical at 522.5 nm. Rods demonstrate peak sensitivity at 495 to 500 nm, whereas green cones demonstrate peak sensitivity at 530 to 540nm. Given the manually directed central foveal stimulation with a stimulus peak energy closer to M-cone peak absorption, the contribution from rods is assumed to be insignificant.

To our knowledge, this is the first report comparing a novel visual sensitivity assay with established conventional visual function tests. IR light is absorbed less by ocular media and therefore penetrates deeper into the eye than visible spectrum light. Traditional visual function tests suffer diminished reliability in patients with lenticular senescence and media opacities.^6^^,^^13^^,^^14^ Ruminski et al.^17^ previously demonstrated greater light scattering and attenuation for 2PM-Vis and less scattering and attenuation for 2PM-IR. Our study confirmed that a series of filters simulating various media opacities and normal aging diminish retinal sensitivity to a greater extent using visible spectrum tests compared to 2PM-IR. A clinical device employing 2PM-IR stimulation would reduce the impact of media opacity on overall visual function and better discern retinal and optic nerve performance.

A limitation in our study evaluating the effects of different transmission filters on retinal sensitivity is our small sample size and limited age range; however, this was intentional to demonstrate the impact of media opacity on visual function tests in young healthy eyes. Brown filters simulating media opacity are most clinically relevant because they mimic cataract. CCT scores were only significantly diminished for the S-cone class and were nominally reduced for M and L-cone classes, which recapitulates other findings by Mehta et al.^20^ It should be noted, however, that CCT, which is very different from microperimetry, and cMP are not scotopic. Both CCT and cMP showed nominal reductions in sensitivity with brown filters. Conversely, 2PM is scotopic, and the effect of brown filters was only significant for 2PM-Vis and not for 2PM-IR. This may suggest that the most sensitive and reliable visual function test is scotopic microperimetry using IR stimulation. Further investigation stratifying 2PM-IR values in different disease states are warranted. Additional ergonomic adjustments to the 2PM will improve ease-of-testing for the subject, especially in diseases causing poor fixation. Automated tracking will facilitate implementation of this technology in a clinical setting to evaluate retinal sensitivity in patients with loss of central fixation.

In conclusion, 2PM-IR can be implemented as a next-generation visual function platform that is nearly impervious to media opacity. This technique would have the greatest utility for studying diseases with a higher prevalence in the aging population, such as glaucoma and age-related macular degeneration.

## References

[1] Kaiser PK Prospective evaluation of visual acuity assessment: a comparison of Snellen versus ETDRS charts in clinical practice (An AOS Thesis). *Trans Am Ophthalmol Soc*. 2009; 107: 311–324. 20126505 PMC2814576

[2] Ahmed SF, McDermott KC, Burge WK, et al. Visual function, digital behavior and the vision performance index. *Clin Ophthalmol*. 2018; 12: 2553–2561. 30573945 10.2147/OPTH.S187131 PMC6292403

[3] Rabin J, Gooch J, Ivan D Rapid quantification of color vision: the cone contrast test. *Invest Ophthalmol Vis Sci*. 2011; 52(2): 816–820. 21051721 10.1167/iovs.10-6283

[4] Rabin J Quantification of color vision with cone contrast sensitivity. *Vis Neurosci*. 2004; 21(3): 483–485. 15518234 10.1017/s0952523804213128

[5] Laishram M, Srikanth K, Rajalakshmi AR, Nagarajan S, Ezhumalai G Microperimetry - a new tool for assessing retinal sensitivity in macular diseases. *J Clin Diagn Res*. 2017; 11(7): NC08–NC11. 10.7860/JCDR/2017/25799.10213 PMC5583893 28892948

[6] Richter-Mueksch S, Sacu S, Weingessel B, Vecsei-Marlovits VP, Schmidt-Erfurth U The influence of cortical, nuclear, subcortical posterior, and mixed cataract on the results of microperimetry. *Eye (Lond)*. 2011; 25(10): 1317–1321. 21738231 10.1038/eye.2011.156 PMC3194310

[7] Hatef E, Hanout M, Moradi A, et al. Longitudinal comparison of visual acuity as measured by the ETDRS chart and by the potential acuity meter in eyes with macular edema, and its relationship with retinal thickness and sensitivity. *Eye (Lond)*. 2014; 28(10): 1239–1245. 25104744 10.1038/eye.2014.182 PMC4194343

[8] Nguyen-Tri D, Overbury O, Faubert J The role of lenticular senescence in age-related color vision changes. *Invest Ophthalmol Vis Sci*. 2003; 44(8): 3698–3704. 12882826 10.1167/iovs.02-1191

[9] Paramei GV, Oakley B Variation of color discrimination across the life span. *J Opt Soc Am A Opt Image Sci Vis*. 2014; 31(4): A375–A384. 24695196 10.1364/JOSAA.31.00A375

[10] Fristrom B, Lundh BL Colour contrast sensitivity in cataract and pseudophakia. *Acta Ophthalmol Scand*. 2000; 78(5): 506–511. 11037903 10.1034/j.1600-0420.2000.078005506.x

[11] Artigas JM, Felipe A, Navea A, Fandino A, Artigas C Spectral transmission of the human crystalline lens in adult and elderly persons: color and total transmission of visible light. *Invest Ophthalmol Vis Sci*. 2012; 53(7): 4076–4084. 22491402 10.1167/iovs.12-9471

[12] Ao M, Li X, Qiu W, Hou Z, Su J, Wang W The impact of age-related cataracts on colour perception, postoperative recovery and related spectra derived from test of hue perception. *BMC Ophthalmol*. 2019; 19(1): 56. 30786855 10.1186/s12886-019-1057-6 PMC6383292

[13] Fujikawa M, Muraki S, Niwa Y, Ohji M Evaluation of clinical validity of the Rabin cone contrast test in normal phakic or pseudophakic eyes and severely dichromatic eyes. *Acta Ophthalmol*. 2018; 96(2): e164–e167. 28556475 10.1111/aos.13495 PMC5836892

[14] Varga A, Sacu S, Vecsei-Marlovits PV, et al. Effect of posterior capsule opacification on macular sensitivity. *J Cataract Refract Surg*. 2008; 34(1): 52–56. 18165081 10.1016/j.jcrs.2007.08.024

[15] Boettner EA, Wolter JR Transmission of the ocular media. *Invest Ophthalmol*. 1962; 1(6): 776–783.

[16] Palczewska G, Vinberg F, Stremplewski P, et al. Human infrared vision is triggered by two-photon chromophore isomerization. *Proc Natl Acad Sci USA*. 2014; 111(50): E5445–E5454. 25453064 10.1073/pnas.1410162111 PMC4273384

[17] Ruminski D, Palczewska G, Nowakowski M, et al. Two-photon microperimetry: sensitivity of human photoreceptors to infrared light. *Biomed Opt Express*. 2019; 10(9): 4551–4567. 31565509 10.1364/BOE.10.004551 PMC6757456

[18] Bowmaker JK, Dartnall HJA Visual pigments of rods and cones in a human retina*.* *J Physiol*. 1980; 298: 501–511. 7359434 10.1113/jphysiol.1980.sp013097 PMC1279132

[19] Konan Medical. ColorDxCCT-HD fundamentals. Available at: https://konanmedical.com/colordx-ccthd-fundamentals/. Accessed March 1, 2020.

[20] Mehta U, Diep A, Nguyen K, Le B, Yuh C, Frambach C, Doan J, Wei A, Palma AM, Farid M, Garg S, Kedhar S, Wade M, Marshall KA, Jameson KA, Cristina KM, Browne AW. Quantifying color vision changes associated with cataracts using cone contrast thresholds. *Trans Vis Sci Tech.* 2020; 0(0): 2612, 10.1167/tvst.0.0.2612. PMC7645251 33200052

